# Long-term neurological outcomes of severe traumatic brain injury in the intensive care unit

**DOI:** 10.3389/fped.2025.1582551

**Published:** 2025-06-23

**Authors:** Bartłomiej Kołodziejczyk, Maria Damps, Karol Żmudka, Marek Mandera

**Affiliations:** Clinical Anesthesiology Department, Faculty of Health Sciences, Medical University of Silesia, Katowice, Poland

**Keywords:** severe traumatic brain injury, pediatric intensive care, cerebral edema, mannitol, long-term neurological outcome

## Abstract

Severe traumatic brain injury (TBI) remains the leading cause of acquired disability in previously healthy children, with outcomes varying widely despite advanced care. Posttraumatic brain damage may prevent proper functioning despite the implementation of advanced intensive care techniques or early neurosurgical interventions. This retrospective cohort study examined the relationship between specific intensive care unit (ICU) interventions and functional outcomes in 69 pediatric patients with severe TBI treated at the Upper Silesian Children's Health Center in Katowice from 2019 to 2024. Data collected included demographics, injury severity, treatment modalities, and intervention procedures. Long-term neurological outcomes were assessed using the Glasgow Outcome Scale (GOS) via parental interviews. The survival rate was 85.5% (59/69), with a median ICU stay of 8 days [interquartile range (IQR) = 5–11]. Of note, the survival rate was significantly longer in boys (8 days, IQR = 6–12.25) than girls (6 days, IQR = 3–9) (*p* = 0.021). Almost all patients (97.1%) required sedation upon admission, with a median mechanical ventilation duration of 6 days (IQR = 4–8). This was also longer among boys (7 days, IQR = 4–9.25) than girls (5 days, IQR = 2–7) (*p* = 0.032). Poorer neurological outcomes (lower GOS scores) were significantly associated with longer ventilation duration (*p* < 0.001), vasopressor administration (*p* = 0.002), transfusion of red blood cells (red blood cell, *p* < 0.001), and transfusion of frozen plasma (fresh frozen plasma, *p* = 0.009). The intubation site did not significantly affect GOS scores (*p* = 0.659). Our findings suggest that pediatric TBI patients requiring prolonged ventilatory support, hemodynamic stabilization, and blood product administration face an increased risk of unfavorable outcomes, highlighting the need for early transfer to specialized pediatric trauma centers to optimize recovery potential. Early referral and access to specialized centers can enhance recovery and improve long-term neurological outcomes.

## 1 Introduction

Traumatic brain injury (TBI) is a leading cause of disability in healthy children. In the European Union, brain injuries result in 57,000 deaths annually and are the main reason for 1.5 million hospitalizations (1). The most common causes of TBIs are traffic accidents and falls (2). The mechanisms of injury have changed over the years, as reflected in research showing that fewer TBIs occur in car accidents than in falls from heights (2, 3). Electric scooters, which can also cause brain damage during accidents, are becoming increasingly popular among all age groups (4). However, all the abovementioned mechanisms of injury are clearly different from war injuries, which are characterized by penetrating or blast TBIs accompanying multiorgan injuries (5, 6). The incidence of TBI in children varies greatly by country, with most reporting rates ranging from 47 to 280 per 100,000 children (7). After the age of 3, boys are more likely to experience TBI than girls. A bimodal age distribution was observed: injuries occurred more often in very young children (0–2 years old) and adolescents (15–18 years old) (7). Mild TBI [Glasgow Coma Scale (GCS) ≥ 13 points] accounts for over 80% of injuries, and up to 90% of all TBIs are related to normal central nervous system (CNS) imaging results. Only a small proportion (<10%) of patients required surgical intervention. Regardless of the country or region of origin, the vast majority of children achieve good neurological outcomes.

According to the literature, children and adolescents have better neurological outcomes following severe TBI than young adults (8). Unfortunately, in low- and middle-income countries, there was twice the risk of death after severe TBI compared with high-income countries, whereas there was no difference in mortality between the mild and moderate TBI groups (9).

Children have a relatively large head compared with the rest of their body, thus significantly shifting their center of gravity and increasing the risk of head injury during an accident. The weaker neck muscles in children than those in adults lead to increased susceptibility to the force of acceleration at the moment of effect. Moreover, the incompletely developed skulls of younger children do not provide adequate brain protection.

TBI can be classified in many ways, such as by type, severity, location, mechanism, and physiological response to injury. Most classification systems are based on patient symptomatology, clinical examination, or diagnostic imaging results in the early phase of stabilization and do not include the evolving process of brain damage. Importantly, the Glasgow Coma Scale (GCS) offers a methodology to objectively assess the state of consciousness after TBI because of its ability to estimate the severity of injury and mortality. Also, computed tomography (CT) has shown that there is an inverse relationship between the GCS score and posttraumatic changes. The need for neurosurgical intervention doubles when the GCS score decreases from 15 to 14 points (10). There is controversy regarding the inclusion of patients with a GCS score of 13 in the mild TBI category because of their higher incidence of intracranial injuries than patients with a GCS of 14–15 (11).

TBI is classified into primary and secondary types on the basis of injury progression. Primary injury is caused by the direct effect of mechanical forces (blunt, penetrating, and explosive) and includes the following: (1) concussion (present symptoms without visible intracranial damage on CT), (2) skull fractures, (3) cerebral contusion (localized local hemorrhages), (4) hematoma (subdural, epidural, intracerebral), (5) subarachnoid hemorrhage, and (6) axonal injury. Secondary injury is a consequence of pathophysiological changes that occur during primary injury and includes the following (12): (1) brain edema, (2) increased intracranial pressure (ICP), (3) further bleeding, (4) seizures, (5) ischemia, (6) infection, and (7) traumatic venous sinus thrombosis.

Due to the serious prognosis after TBI, treatment is important to limit health consequences in these patients. This study aimed to assess the effect of treatment and interventional procedures on long-term neurological outcomes. We aimed to identify positive prognostic factors to improve trauma management. There is a lack of data on how specific interventions influence long-term neurological outcomes in pediatric patients with severe TBI. This study aimed to bridge this knowledge gap by analyzing the outcomes in a single-center pediatric intensive care unit (ICU) over 5 years. Our work aimed not only to present the long-term results of severe TBI treatment but also to present in detail the elements of complex and multifactorial treatment in the ICU. Researchers have presented a procedure model developed in an academic center and showed that the use of this model led to good neurological outcomes in half of the studied patients, thus allowing critically ill children to return to normal functioning in society.

## 2 Methods

This study was conducted at the Department of Anesthesiology and Intensive Care at the Upper Silesian Children's Health Center in Katowice. Data were collected from children hospitalized due to severe TBI in 2019–2024. A database was created using hospitalization data, considering age, sex, days of ICU stay, days of mechanical ventilation, circulatory system support, sedation, analgesia, used antibiotics, therapy to reduce brain swelling, neurosurgery intervention, ICP sensor implantation, RBC/FFP transfusion, pro-cognitive treatment, intubation site, reintubation after extubation in ICU, discharge department, death, organ donation.

The children we treated were mostly victims of road accidents and falls from heights, but detailed information on the mechanisms of injury was not recorded in the documentation provided by the emergency medical teams.

The study was designed as a retrospective–prospective cohort study. The inclusion criterion was severe traumatic brain injury in children, and the exclusion was brain damage not caused by trauma. We used the Glasgow Coma Scale (GCS) to objectively assess the state of consciousness after TBI because of its ability to estimate the severity of injury and mortality (Table 1). Long-term treatment results were assessed one year after discharge by an anesthesiologist via telephone contact with the patient's parents by using the Glasgow Outcome Scale (GOS). The primary outcome of the study was surviving ICU hospitalization, andthe secondary outcomes included functional status, assessed based on parental report, focusing on the child's ability to perform age-appropriate daily activities.

**Table 1:** Glasgow Coma Scale.

Glasgow Coma Scale (GCS)		
Best eye response (4 points)	Best verbal response (5 points)	Best motor response (6 points)
1. No eye opening 2. Eye opening to pain 3. Eye opening to sound 4. Eyes open spontaneously	1. No verbal response 2. Incomprehensible sounds 3. Inappropriate words 4. Confused 5. Orientated	1. No motor response 2. Abnormal extension to pain 3. Abnormal flexion to pain 4. Withdrawal from pain 5. Localizing pain 6. Obeys commands
Relation between TBI and GCS		
Mild TBI: GCS 13–15; mortality 0.1% Moderate TBI: GCS 9–12; mortality 10% Severe TBI: GCS <9; mortality: 40%		

The results obtained were subjected to statistical analyses. We reported the medians and interquartile ranges (IQRs) for continuous variables and the number of cases with proportions for categorical variables. We used the Mann–Whitney *U* test and Kruskal–Wallis test to compare data between two non-normally distributed independent groups and three independent groups, respectively. The categorical variables were analyzed using the chi-squared test. For all analyses, two-sided *p* values of ≤0.05 were considered statistically significant. All statistical analyses were performed using RStudio statistical software (2,023.09.0 + 463).

## 3 Results

**Figure 1:**
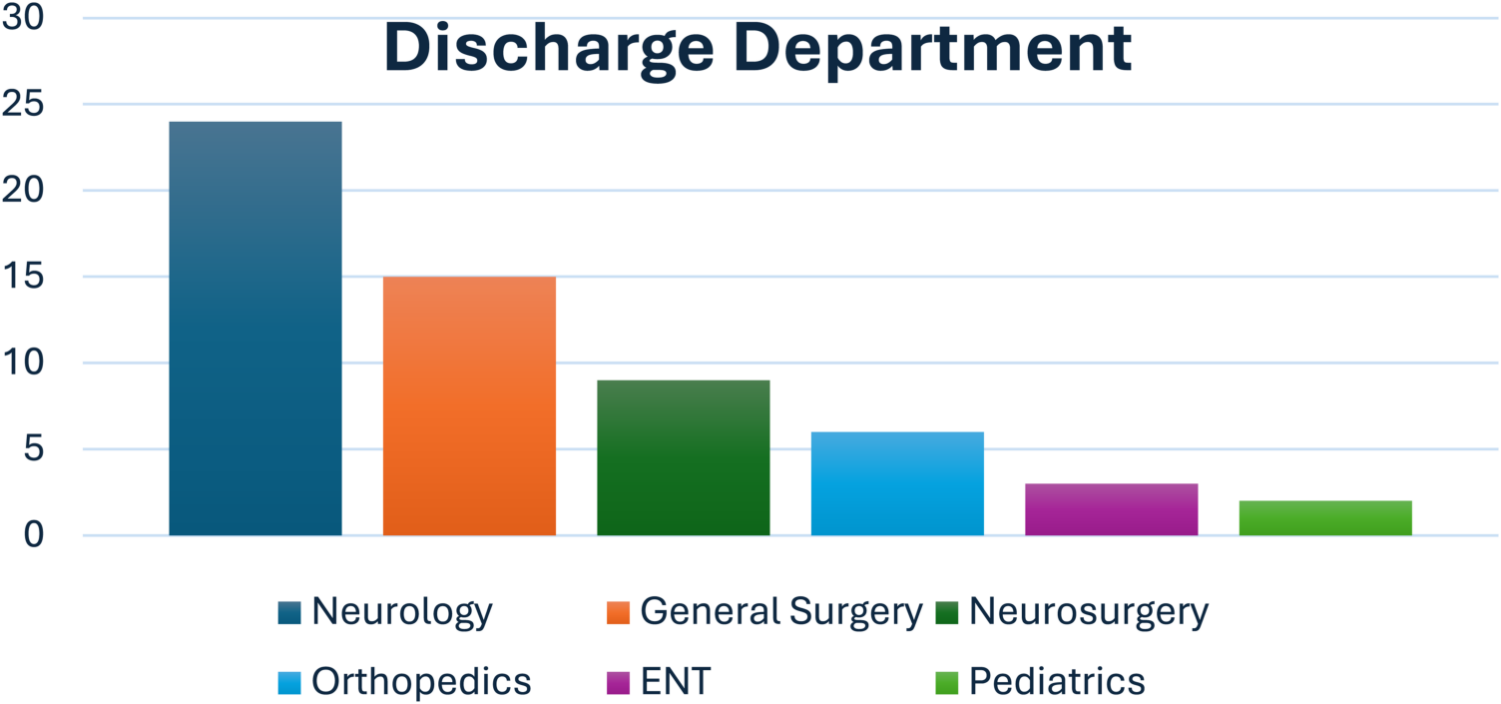
Percentage distribution of departments to which children were transferred to after discharge.

In the Department of Anesthesiology and Intensive Care of the Upper Silesian Children's Health Center in Katowice, 69 children were hospitalized due to severe TBI between January 2019 and February 2024. This group comprised 40 boys (57.97%) and 29 girls (42.03%). The median hospitalization time was 8 days (IQR = 5–11) and was significantly longer in boys (8 days, IQR = 6–12.25) than that in girls (6 days, IQR = 3–9) (*p* = 0.0202), with the shortest hospitalization time being 1 day and the longest being 75 days. Ten (14.49%) deaths were recorded at hospital discharge. Among those who died, six (60%) were males, and four (40%) were females. Brain death was declared by the committee for five children (50%; three males and two females), all of whom were included in the organ donation program. The patients were transferred from the ICU to different departments for further treatment (Figure 1).

After discharge, two children (2.9%) required rehospitalization: a 17-year-old boy with severe hospital-acquired pneumonia and a 15-year-old girl with neuro-infection after functional endoscopic sinus surgery. We also obtained information on two additional deaths after hospital discharge, one due to a successful suicide attempt and the other due to severe acute respiratory syndrome (coronavirus 2 infection). ICU treatment included the continuation of mechanical ventilation, sedation, analgesia, reduced brain swelling, circulatory system support, antibiotics, neurosurgical intervention, blood product transfusion, and precognitive and antipsychotic treatments. The elements of ICU therapy and their associations with mortality and GOS scores were analyzed. The observed relationships are detailed below.

### 3.1 Intubation

Most patients (*n* = 31) were intubated at the accident site by air ambulance personnel; the remaining intubation sites are listed below (Table 2). We compared the GOS scores among the three groups by using the Kruskal–Wallis test and found no significant differences (*p* = 0.6594).

**Table 2:** Intubation site.

Intubation site	*n* (%) of patients
Accident site	38 (56.7%)
Accident site—air ambulance	31 (46.3%)
Accident site—ambulance	7 (10.4%)
Emergency department	26 (37.68%)
Our emergency department	11 (16.4%)
Another emergency department	15 (22.4%)
Operating theater	3 (4.5%)

### 3.2 Mechanical ventilation

Mechanical ventilation was continued in 67 (97.1%) patients. The median ventilation time was six days (IQR = 4–8) and was longer among boys (7 days, IQR = 4–9.25) than that among girls (5 days, IQR = 2–7) (*p* = 0.0315). Patients who died shortly after admission or who were awakened and extubated were ventilated for the shortest time (1 day). A 14-year-old girl was ventilated for 45 days, and her stay lasted for 75 days. After extubation, five patients (74.6%) required reintubation because of acute respiratory failure caused by subglottic swelling. The most frequently used mechanical ventilation mode for maintaining normocapnia was adaptive support ventilation (ASV).

### 3.3 Sedation

On admission, 67 patients (97.1%) were sedated to a Richmond Agitation and Sedation Scale (RASS) score of −5. In most cases, the assessment of neurological conditions using the GCS scale at the accident site was not recorded in the documentation by emergency medical teams. A RASS score of −5 was used (Table 3). Masimo Root with O3 Regional Oximetry platform was routinely used to monitor the depth of sedation [electroencephalography (EEG)] and cerebral oxygenation level (rSO_2_) by using near-infrared spectroscopy (NIRS). Ketamine was used in cases of extreme circulatory system failure requiring a supply of pressor amines to maintain cerebral perfusion pressure (CPP).

**Table 3:** Sedation.

Sedation	*n* (%) of patients
Thiopental	62/69 (89.8%)
Propofol	32/69 (46.3%)
Dexmedetomidyne	30/69 (43.5%)
Ketamine	2/69 (2.9%)

### 3.4 Analgesia

Several analgesic methods were used in the patients (Table 4). In most cases, TBI coexists with other injuries. Continuous sufentanil infusion provided effective analgesia, and nalbuphine boluses were most commonly administered after completion.

**Table 4:** Analgesia.

Analgesia	*n* (%) of patients
Sufentanil	54/69 (78.3%)
Nalbuphine	17/69 (24.6%)
Fentanyl	7/69 (10.1%)
Morphine	1 (1.4%)
Remifentanyl	1 (1.4%)

### 3.5 Therapy to reduce brain swelling

The standard feature was a headrest raised at 30°. Pharmacotherapy was based on mannitol boluses (2 g/kg body weight, administered four times a day). Mannitol was administered to 42 patients, whereas 17 did not receive it. We compared the GOS scores of patients who received mannitol with those of patients who did not. The median GOS score was 5 (IQR = 3–5) among those who received mannitol, and 5 (IQR = 3.25–5) among those who did not receive it. The difference between the groups was not statistically significant (*p* = 0.7768664).

### 3.6 Circulatory system support

Several pressor amines were used to maintain CPP (Table 5). Of note, all patients who required an infusion of epinephrine or milrinone died.

**Table 5:** Pressor amines.

Pressor amines	*n* (%) of patients
Norepinephrine	30/69 (43.5%)
Dopamine	7/69 (10.1%)
Epinephrine	6/69 (8.7%)
Milrinone	1/69 (1.4%)

### 3.7 Antibiotic therapy

Upon admission to the ICU, 64 patients (92.7%) received empirical antibiotic therapy, and only 11 (15.9%) received therapy that was modified to the target. The most frequently used antibiotic was ceftriaxone, followed by amoxicillin and clavulanic acid. Due to the limited number of cases, we only compared GOS levels between those who received ceftriaxone [median GOS score of 4 (IQR = 3–5)] and those who received amoxicillin + clavulanic acid [median GOS score of 5 (IQR = 4–5)]. No significant differences were found (*p* = 0.4998) (Table 6).

**Table 6:** Antibiotic therapy.

Antibiotic therapy	Number of patients
Ceftriaxone	39
Ceftriaxone + clindamycin	4
Cefuroxime	2
Amoxicillin + clavulanic acid	15
Amoxicillin + clavulanic acid + clindamycin	1
Piperacillin + tazobactam	3

### 3.8 Neurosurgical intervention

Fifteen patients (21.7%) required neurosurgical intervention (12 craniotomies) to remove intracranial hematoma. We found that the median GOS scores among those who underwent and did not undergo neurosurgical intervention were 4 (IQR = 1–5) and 5 (IQR = 3–5), respectively. The difference in GOS scores between the groups was significant (*p* = 0.001781). Moreover, five patients required ICP sensor implantation (three of which required only surgical intervention). We found that the median GOS score among those who received and did not receive the ICP sensor was 3 (IQR = 1–5) and 5 (IQR = 3–5), respectively. The difference in GOS scores between the groups was not significant (*p* = 0.3348).

### 3.9 Remaining treatments

A total of 22 patients (31.9%) required red blood cell (RBC) transfusion, and 14 patients (20.3%) required fresh frozen plasma (FFP). Antipsychotic treatment was initiated in eight (11.59%) children and continued after discharge. After hospitalization, we asked parents about their children's current functioning level in society. Information was obtained from 52 caregivers (75.36%). The assessment was performed using the five-point GOS (13–15) (Table 7). We also compared the applied treatment and intervention procedures associated with better long-term treatment results.

**Table 7:** Glasgow Outcome Scale.

GOS	Good recovery	Moderate disability	Severe disability	Persistent vegetative state	Death
Scoring	5	4	3	2	1
Number of patients	29	6	5	0	12

**Figure 2:**
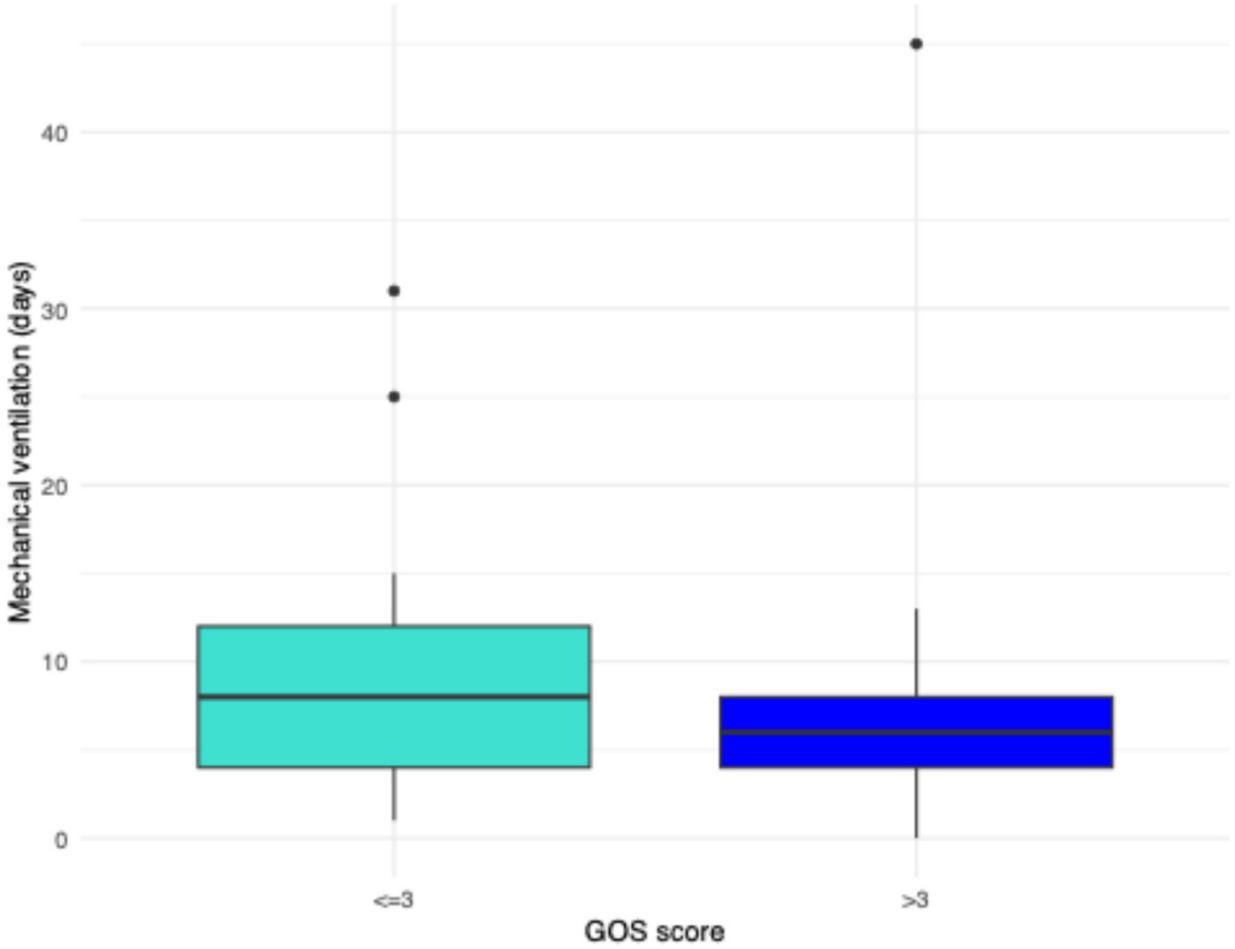
Duration of mechanical ventilation in relation to Glasgow Outcome Scale (GOS) score. Box plots show the number of days on mechanical ventilation for patients with poor neurological outcomes (GOS ≤ 3, turquoise) and favorable outcomes (GOS > 3, blue). The boxes represent the interquartile range (IQR), the horizontal line within each box indicates the median, and the whiskers extend to 1.5 times the IQR. Dots represent outliers.

**Figure 3:**
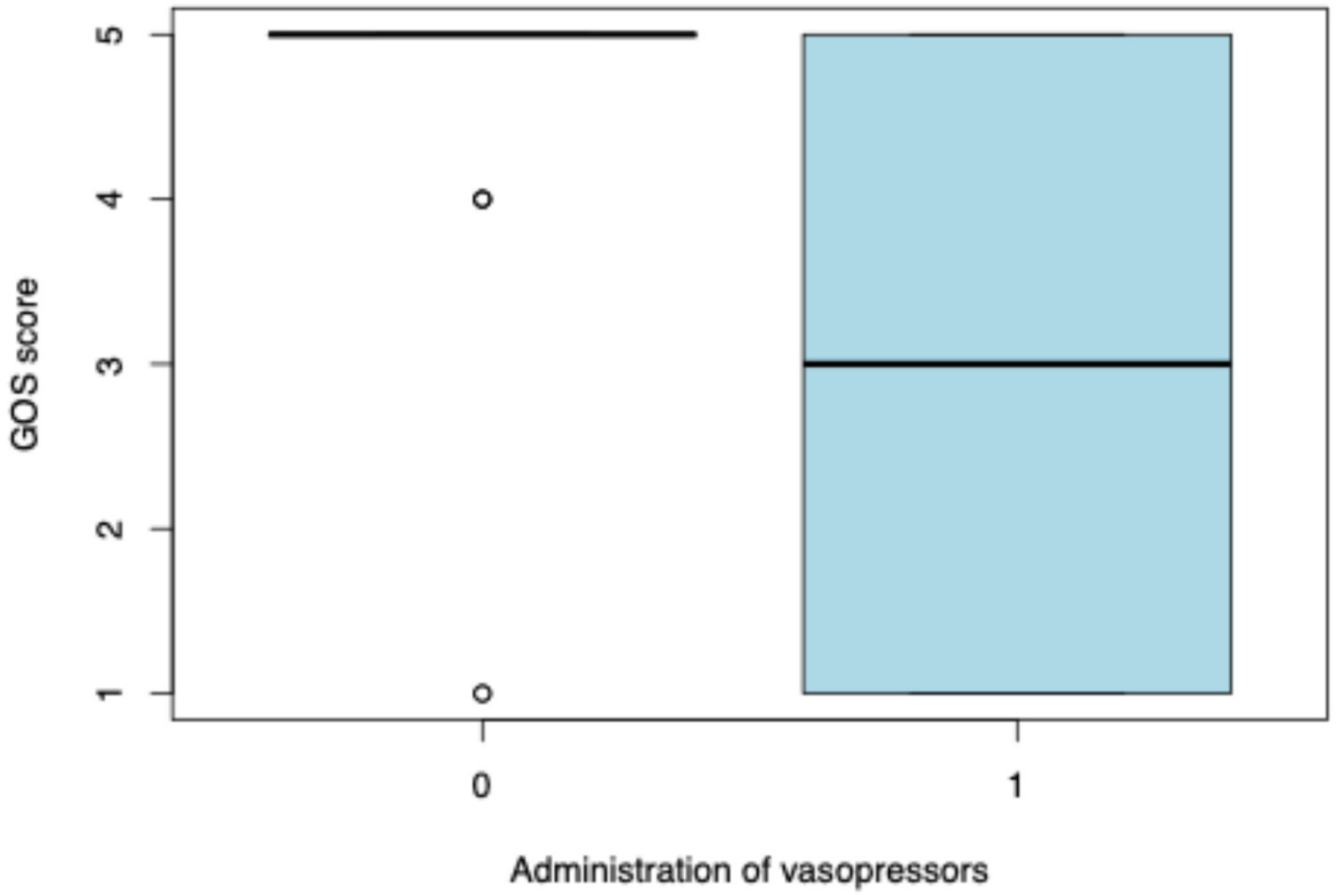
Association between vasopressor administration and Glasgow Outcome Scale (GOS) score. The box plots illustrate GOS scores in patients who did not receive vasopressors (0) and those who did (1). The boxes represent the interquartile range (IQR), the horizontal line inside each box indicates the median, and the whiskers extend to 1.5 times the IQR. Circles represent outliers.

**Figure 4:**
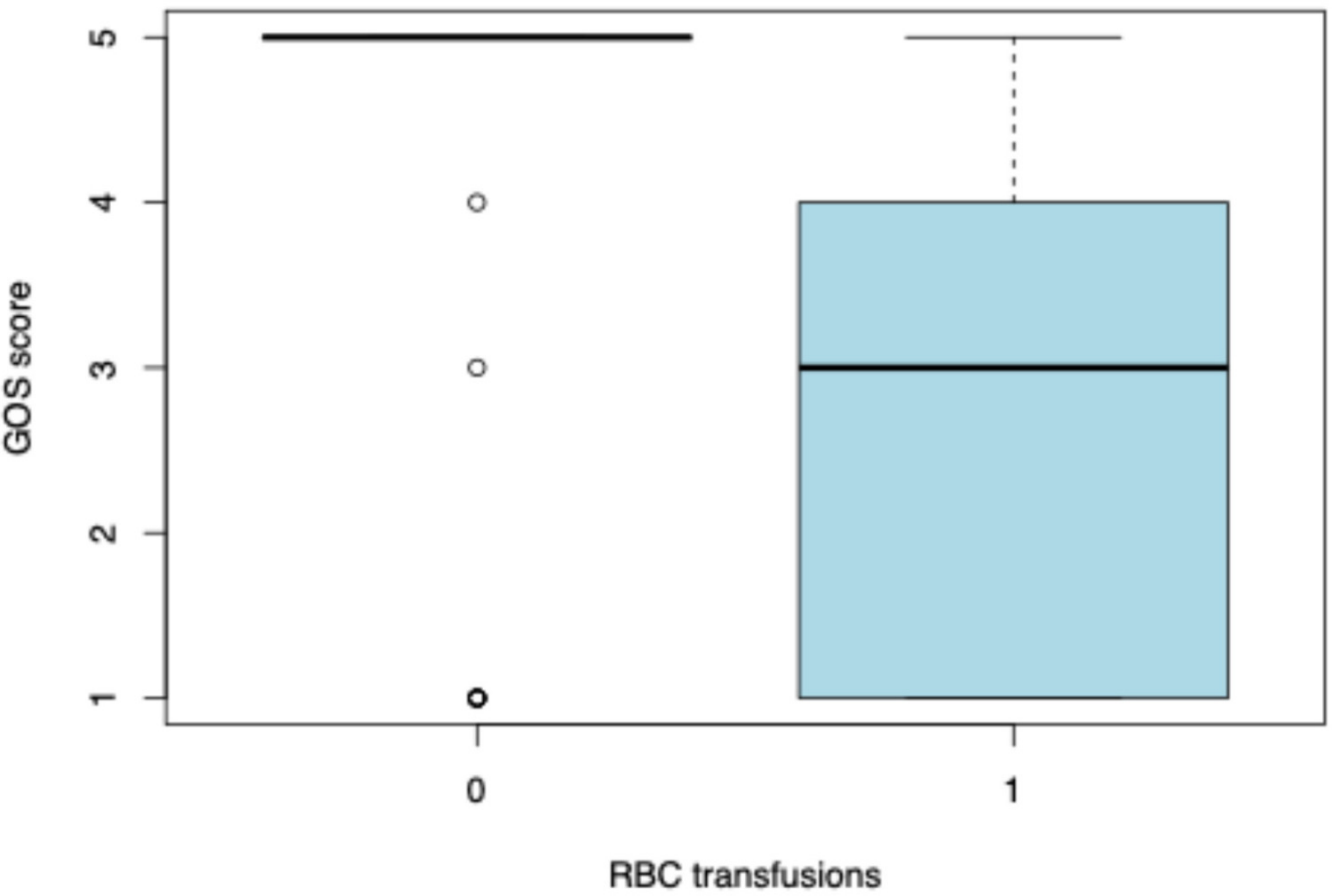
Glasgow Outcome Scale (GOS) scores in relation to red blood cell (RBC) transfusions. The box plots compare GOS scores between patients who did not receive RBC transfusions (0) and those who did (1). The boxes represent the interquartile range (IQR), the bold horizontal line within each box indicates the median, and the whiskers extend to 1.5 times the IQR. Open circles represent statistical outliers.

**Figure 5:**
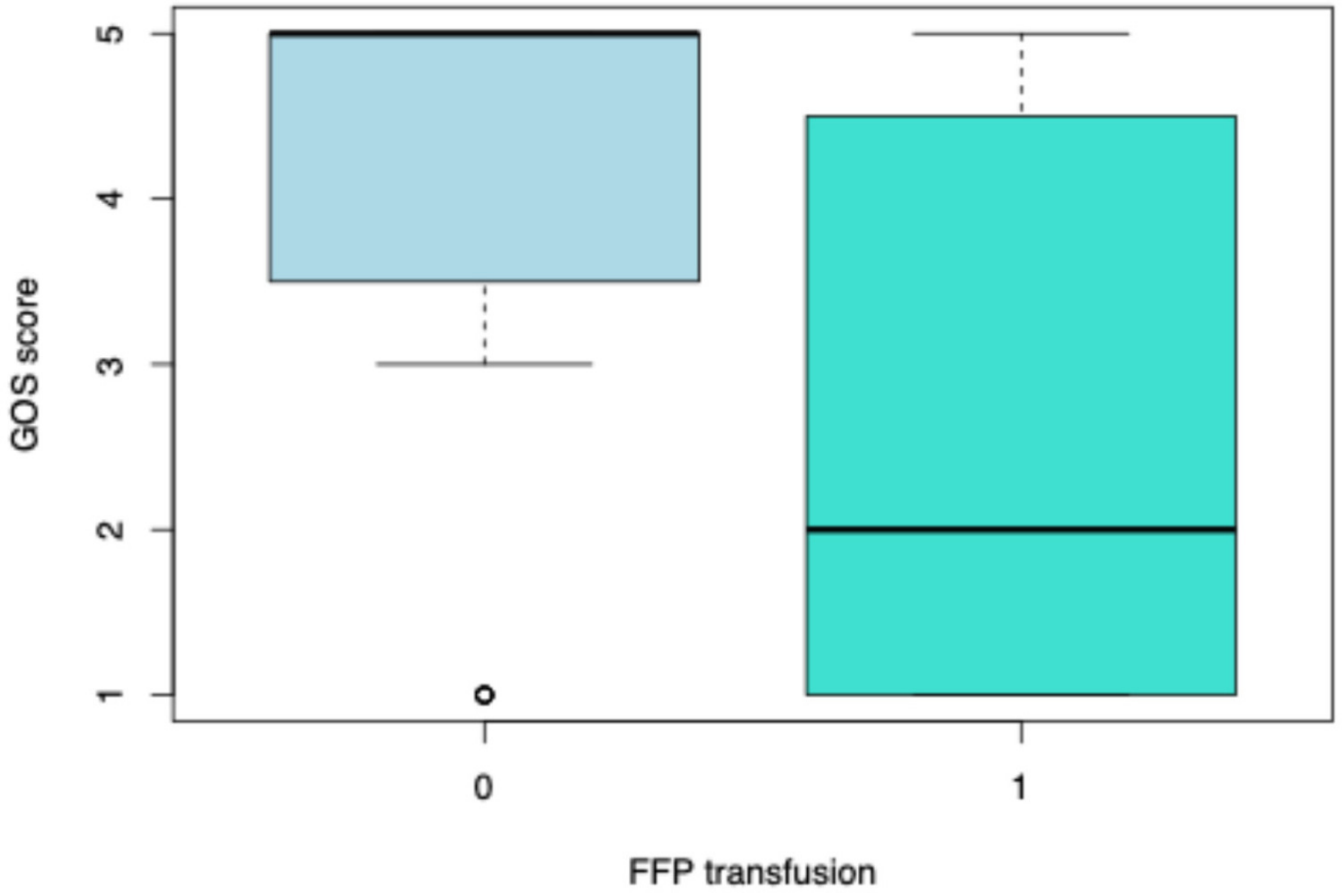
Glasgow Outcome Scale (GOS) scores in relation to FFP transfusions. The box plots compare GOS scores between patients who did not receive FFP transfusions (0) and those who did (1). The boxes represent the interquartile range (IQR), the bold horizontal line within each box indicates the median, and the whiskers extend to 1.5 times the IQR. Open circles represent statistical outliers.

We analyzed the factors that were associated with higher GOS scores. We observed that a lower GOS score was associated with a longer ventilation duration (*p* < 0.0001) (Figure 2). Moreover, patients who were administered vasopressors had a lower median GOS score (GOS = 3, IQR = 1–5) than that of those who did not receive them (GOS = 5, IQR = 5–5) (*p* = 0.001781) (Figure 3). Lower GOS scores were observed in patients who received RBC transfusions (GOS = 3, IQR = 1–4) than those in patients who did not receive RBC transfusions (GOS = 5, IQR = 5–5) (*p* = 0.0006) (Figure 4). Additionally, lower GOS scores were observed in patients who received FFP (GOS = 2, IQR = 4–1) than those of patients who did not receive FFP (GOS = 5, IQR = 3.5–5) (*p* = 0.009) (Figure 5).

## 4 Discussion

The multimodal treatment of pediatric patients after severe TBI is aimed at maintaining CPP at an appropriate physiological value. To achieve this, one should strive for normal arterial blood pressure and minimize elevated ICP (16). Adequate brain perfusion helps maintain proper blood oxygenation and prevents hypoxia. The important criteria for treatment success include the severity of injury and the period when cerebral flow and perfusion are disrupted. The results of our study confirmed the positive correlation between a patient's serious condition at the time of injury and the need for prolonged multisystem support, including analgosedation.

At our center, the most commonly used anesthetic is thiopental, which reduces the metabolism of damaged brain tissue (CMRO_2_) to the same extent as propofol; however, its infusion requires more frequent use of pressor amines (17). According to the guidelines of the US Food and Drug Administration, the prolonged continuous infusion of propofol for sedation or management of refractory intracranial hypertension is not recommended (16). The pediatric population is particularly at risk of propofol-related infusion syndrome, which leads to multiple organ failure and death (18). The addition of sufentanil is an effective method of analgesia in multiple organ injuries. Compared with fentanyl, sufentanil shortens the return time to spontaneous breathing and ICU stay (19). Sufentanil also has no effect on ICP and blood flow velocity in the CNS (20).

More than 97% of children required invasive respiratory support. During mechanical ventilation, it is crucial to maintain pCO_2_ within 35–40 mmHg to ensure proper blood flow through the CNS vessels. The only indication for temporary hyperventilation and pCO_2_ reduction is threatening cerebral herniation (16), but this intervention is associated with the risk of cerebral tissue ischemia. At our center, the most frequently chosen mode is ASV, in which it is possible to simultaneously correlate arterial blood gas (ABG) results with end-tidal carbon dioxide (etCO_2_) and for the target values to be set by the physician. The Guidelines for the Management of Severe TBI (Third Edition; Brain Trauma Foundation, 2019) (16) in children suggest the use of ICP monitoring; however, this is a level III recommendation (based on poor quality scientific evidence). In the current study, the ICP sensor was implanted in 7.2% of the patients, which corresponded to a global average of 7.7% (21). Alkhoury and Kyriakides (21) and Bennett et al. (22) observed that the final treatment results were better in children whose ICP was monitored, although these patients were ventilated and hospitalized longer probably due to the severity of the injury and the need for prolonged therapy.

As previously mentioned, the goal of therapy is to maintain CPP (at least 40 mmHg) despite an elevated ICP. One of the methods for reducing ICP is the infusion of mannitol or hypertonic saline. Currently, there is an ongoing discussion on and against a given osmotically active agent. Kochanek et al. (23) compared the use of hypertonic saline and mannitol; both agents had the same effect on maintaining CPP with elevated ICP.

Electrolyte disturbances can complicate the course of treatment. Hypernatremia, which is defined as Na+ concentration > 160 mmol/L, is associated with a mortality rate of 75% (24). However, hyponatremia, which is defined as Na+ concentration < 135 mmol/L, is an independent risk factor for poor neurological outcomes (25). Therefore, our patients usually undergo ABG tests twice a day or more if electrolyte values are borderline. In addition, the fluid balance was calculated every 6 h to ensure euvolemia.

To maintain CPP, it is critical to combat hypotension, which increases mortality and is caused by sedation and systemic inflammatory response syndrome due to trauma. The use of catecholamines, mainly norepinephrine, to constrict the vascular bed (26) was required in over 60% of the analyzed patients. The proper volume of the vascular bed is also important for maintaining normal blood pressure. Blood derivatives were used in every second patient.

In addition to intensive treatment aimed at ensuring CPP, we performed regular CT scans of the head, particularly on the second and third days after injury, which are the days when vasospasm typically develops, blood flow through the CNS is limited (27), and the risk of ischemia increases. A more severe injury leads to a higher risk of ischemia (28), which persists for many years after the end of hospitalization. The Brain Trauma Foundation recommendations for performing head CT do not specify time intervals, but neuroimaging is reasonable in cases of deterioration of the neurological condition and increased ICP (level III recommendation).

In our opinion, there is a need to create a unified system of support for patients and their families after discharge from the ICU to continue comprehensive treatment in post-intensive care syndrome (PICS) follow-up clinics by a team of specialists consisting of a doctor, nurse, physiotherapist, dietitian, psychologist, and pharmacist. PICS follow-up clinics are optimal solutions; however, home visits, telemedicine, support groups, and planned specialist consultations are also possible (29). It would be best if there were a separate function for the care coordinator after discharge from the ICU.

The long-term assessment of clinically discharged patients is of great value. Half of the patients obtained the maximum GOS scores. To our knowledge, this is the first study of this type in Poland to assess the clinical management and survival of children with TBI. Researchers did not focus on just one selected element of the therapy but combined the entire treatment process into one coherent whole.

One limitation of our study was the lack of information regarding the patients' conditions at the accident site and the time until qualified medical assistance arrived. First, this was a retrospective study. We did not have a control group to compare the activities that significantly translated into improved survival. The analysis was performed at one center, which is another limitation of this study. It is necessary to conduct further prospective studies that use all clinical data from the moment of injury, including its mechanism, and the activities undertaken in the prehospital period. Furthermore, national guidelines for the management of TBI in children should be developed.

### 4.1 Clinical implications/future directions

Our study highlights the need to individualize the treatment of children with severe TBI to achieve good neurological outcomes and enable a return to normal functioning. In summary, our treatment consists of the following: (1) analgosedation for RASS “−5” (sufentanil + thiopental), (2) mechanical ventilation in ASV mode, (3) circulatory support with norepinephrine, (4) advanced brain function monitoring (EEG + NIRS), (5) therapy to reduce brain swelling, (6) empirical antibiotic therapy (ceftriaxone), and (7) control CT scan of the head for 24 h and up to 3 days after injury for the re-evaluation and early diagnosis of vasospasm. Multicenter studies are required to confirm the management strategies used at our center.

## 5 Conclusions

Lower GOS scores were associated with longer mechanical ventilation time, pharmacological support of the circulatory system, and transfusion of blood products.

## Data Availability

The raw data supporting the conclusions of this article will be made available by the authors, without undue reservation.
