# Supplementary material for: Long-term neurological outcomes of severe traumatic brain injury in the intensive care unit
Source: Front Pediatr. 2025 Jun 23;13:1582551. doi: 10.3389/fped.2025.1582551 (PMC12230999; doi:10.3389/fped.2025.1582551)
Supplement: Supplementary file 1 [file Datasheet1.docx]

The Glasgow Coma Scale divides into three parameters: best eye response (E), best verbal response (V) and best motor response (M).

*Best eye response (4 points)*

1. No eye opening
2. Eye opening to pain
3. Eye opening to sound
4. Eyes open spontaneously

*Best verbal response (5 points)*

1. No verbal response
2. Incomprehensible sounds
3. Inappropriate words
4. Confused
5. Orientated

*Best motor response (6 points)*

1. No motor response.
2. Abnormal extension to pain
3. Abnormal flexion to pain
4. Withdrawal from pain
5. Localizing pain
6. Obeys commands
